# Social connection and living with severe chronic obstructive pulmonary disease: A qualitative analysis

**DOI:** 10.1177/13591053251412948

**Published:** 2026-01-25

**Authors:** Lisa Jane Brighton, Katherine Bristowe, Catherine Evans, Morag Farquhar, William D.-C. Man, Margaret Ogden, Andy Phillips, Matthew Maddocks, Joseph Chilcot

**Affiliations:** 1 King’s College London, UK; 2 Sussex Community NHS Foundation Trust, Brighton, UK; 3 University of East Anglia, Norwich, UK; 4 Guy’s and St Thomas’ NHS Foundation Trust, London, UK; 5 Imperial College London, National Heart and Lung Institute, UK; 6 Cicely Saunders Institute Public Involvement Group, London, UK

**Keywords:** chronic obstructive pulmonary disease, qualitative research, social support, social isolation, loneliness, biopsychosocial model

## Abstract

People with chronic obstructive pulmonary disease (COPD) face increased risk of social isolation and loneliness. However, social dimensions are frequently overlooked in respiratory care. We aimed to explore the role of social connection in living with COPD, including influences on health and function. We conducted a reflexive thematic analysis of semi-structured interviews with 19 people with COPD (median age 78 years [range 58–88]; 14 with severe airflow obstruction). Three themes were identified: social connection supports COPD self-management, the “triple threat” of COPD to social connection, and the inseparable nature of social health. Participants described how worsening symptoms, particularly breathlessness, contribute to disconnection through physical restrictions, psychological reactions, and societal unawareness, with negative impacts on self-management and wider physical and psychological health. We conclude that social connections become increasingly valuable, yet increasingly difficult to maintain, as COPD progresses. Supporting individuals to maintain connections within a biopsychosocial approach may unlock wider health benefits.

## Introduction

Chronic Obstructive Pulmonary Disease (COPD) is an incurable condition that affects an estimated 480 million people worldwide (Boers et al., 2023). Over 1 million people in the UK have a diagnosis of COPD, with a further estimated half million people living with the condition undiagnosed (Stone et al., 2023). Common and disabling symptoms include breathlessness, cough, and fatigue, which tend to worsen over time. Treatments and supported self-management strategies are available to help people with COPD live as well as possible with their condition. However, many still experience psychological distress (Volpato et al., 2023) and persistent limitations in daily activities (Gardener et al., 2018). Perhaps related to this, recent population-based evidence has shown that people with COPD are at greater risk of social isolation and loneliness (Suen et al., 2023), report a reduced sense of belonging, and struggle to rely on others when experiencing high levels of stress (Mete, 2024).

Evidence predominantly from the United States suggests the pooled prevalence of loneliness in people with COPD is 32% (95% CI = 16%–48%, *K* = 4) (Alqahtani et al., 2024), while a nationally representative cohort study found one in six with COPD reported social isolation (Suen et al., 2023). Several factors might contribute to these challenges with social connection. Across quantitative studies (Gardener et al., 2019; Marty and Benzo, 2019; Michalovic et al., 2021), qualitative studies (Brighton et al., 2020; Schroedl et al., 2014), and systematic reviews (Disler et al., 2014; Gardener et al., 2018; Hutchinson et al., 2018; Lovell et al., 2019) exploring the lived experiences of people with COPD, participants have reported facing a shrinking social world due to the impacts of their symptoms and treatments (e.g. portable oxygen) on mobility and participation. This can be further compounded by societal issues of stigma and shame due to perceptions of their illness as “self-inflicted” due to associations with smoking (Gysels et al., 2016), and more recently negative public reactions to symptoms like cough due to associations with Covid-19 (Mathioudakis et al., 2021).

Despite the multidimensional impacts of COPD indicating the need for a biopsychosocial approach, and the potential benefits for furthering self-management, promoting person-centredness, and reducing psychological distress (Gonçalves et al., 2025), dedicated consideration of social elements is often missing from COPD research and care. This phenomenon is not new: the “social” in biopsychosocial has historically been neglected compared to its biological and psychological elements (Barkley, 2009; Suls and Rothman, 2004). While there has been some qualitative exploration of the role of stigma in COPD, there is a dearth of studies explicitly focused on social connection. This is surprising, given known adverse impacts of social disconnection on health (Holt-Lunstad et al., 2015; Park et al., 2020), and the numerous times people with COPD have expressed this as an area of concern as part of broader studies (Disler et al., 2014; Gardener et al., 2018; Hutchinson et al., 2018; Lovell et al., 2019). The reverse is also true in the context of COPD, with higher levels of social support being linked to improved quality of life, mental health, and functional outcomes, but also less severe COPD (Aravantinou-Karlatou et al., 2023).

Understanding how social connections contribute to wider health in the context of respiratory illness is essential for effective, person-centered, holistic interventions. As people with COPD tend to live with multiple long-term conditions (Divo et al., 2018), this work may also provide insights relevant to supporting people with other chronic illnesses. To inform a complete biopsychosocial approach to respiratory research and care, this analysis aims to explore the role of social connections in living with COPD. Within this context, social connection is defined as “connections that exist between people who have recurring interactions that are perceived by the participants to have personal meaning” (August and Rook, 2013: 1838). Our objectives are to explore how social connections contribute to the lived experience of COPD, their impacts on health and function, and how they are influenced by individual, inter-personal, and societal factors.

## Methods

### Design

Supplementary secondary qualitative analysis (Heaton, 2008).

### Procedure

Full details for the original qualitative interview study are reported elsewhere (Brighton et al., 2020). In brief: the original study aimed to understand experiences of people with both COPD and frailty referred for outpatient pulmonary rehabilitation. Participants were identified by clinical staff during their initial assessment for outpatient pulmonary rehabilitation at two London hospitals, and were eligible if they had a physician diagnosis of COPD and a Short Physical Performance Battery (Guralnik et al., 1994) score of ⩽9, indicating pre-frailty or frailty. People under the age of 18 years, unable to speak English, or without mental capacity to provide informed consent were excluded. Of those eligible, participants were purposively sampled for diversity in age (>/⩽ 80 years), living status (alone/ with others), level of physical frailty (SPPB scores of >/⩽7), and completion of pulmonary rehabilitation (did/did not complete). If preferred by the participant, unpaid/family caregivers could also participate alongside them.

Semi-structured interviews were conducted by a researcher (LJB) with a background in psychology and palliative care research. The interviewer was not known to participants prior to the interviews. The topic guide was developed in collaboration with stakeholders with relevant professional and lived experience, drawing on theories of aging (Baltes and Baltes, 1990), self-regulation (Leventhal et al., 2016), and coping (Lazarus and Folkman, 1987). Questions explored experiences of pulmonary rehabilitation, participants’ current health and priorities, support they were receiving, and unmet needs. Most of the interviews took place in participants’ homes (*n* = 17), two took place at the researcher’s university (Brighton et al., 2020). In three interviews, participants were accompanied by a family member: two who consented for their contributions to be included, one who was present but did not participate. Data collection ceased when preliminary analysis suggested thematic saturation and sufficient information power.

### Analysis

We reanalyzed the pseudonymized transcripts using reflexive thematic analysis (Braun et al., 2018; Braun and Clarke, 2019), with reference to the original field notes. All transcripts from the original study were included, as all contained references to social connection. One researcher (LJB) inductively generated codes and themes, with guidance from KB. Initial coding was completed by hand on paper copies of the transcripts, and then organized and applied in NVivo 14 (Lumivero, 2023). Codes and themes were initially organized in a table. The team moved between digital tools (e.g. exploration via NVIVO queries) and manual approaches (e.g. visual mapping), to iteratively refine the codes and themes until the final interpretation was agreed.

The themes were reviewed and refined with the wider project team, which includes people from diverse professional backgrounds (e.g. nursing, physiotherapy, psychology), and people personally affected by COPD as a patient or unpaid/family carer. Themes were also refined through engagement with relevant theoretical work: we drew on Holt-Lunstad’s model of direct and indirect pathways linking social connection and morbidity/mortality (Holt-Lunstad, 2021), a conceptual model of social dimensions of respiratory disease (Brighton et al., 2022), and the Social Relationship Expectations Framework (Akhter-Khan et al., 2023).

Holt-Lunstad’s (2021) model summarizes how the structure, function and quality of social connection links to behavioral, biological and psychological factors contributing to morbidity and mortality. It is based on a review of evidence across health sciences including from people with long-term conditions. We drew on this model to consider the interrelations between social and other dimensions of health and potential mechanisms by which these occur. Brighton et al.’s (2022) model reflects evidence of how a subset of these interrelationships—specifically between loneliness, social isolation, stigma, and poorer health outcomes—manifest in the context of chronic respiratory disease. Akhter-Khan et al’s Social Relationship Expectations Framework (2023) is based on evidence from across psychology, gerontology, and anthropology. It outlines four universal relationship expectations (proximity, support, intimacy, and fun), and two that are deemed age-specific: respect and generativity. Given the context of our sample, who are experiencing aging and functional limitation, we referred to this framework to consider these dimensions during our analysis.

In line with the original qualitative study, we approached this analysis within a critical realist paradigm (Fletcher, 2017) that acknowledges how social and cultural structures influence empirical explorations of reality. LJB engaged with reflexive practice through analytical journaling and discussing her thinking with wider members of the team. LJB had been motivated to conduct the secondary analysis based on discussions with people affected by COPD and having noticed the prominence of stories about social connection when conducting the interviews and primary analysis. Given LJB felt highly familiar with the data, presenting preliminary findings and illustrative data to team members was an important part of ensuring analytical rigor and keeping interpretation rooted in participants’ accounts.

### Ethics

The London Camberwell St Giles Research Ethics Committee (ref. 18/LO/1197) approved the original study. All participants consented to future analysis of their anonymized data.

## Results

The 19 participants were mostly living with severe lung obstruction and multiple long-term conditions; further participant details are shown in Table 1. Eleven of the participants lived alone. All participants discussed some element of social connection that was relevant to the secondary analysis. Three themes were constructed: (1) social connection supports COPD self-management; (2) the “triple threat” of COPD to social connection; and (3) the inseparable nature of social health.

**Table 1.** Participant Characteristics (*n* = 19; Brighton et al., 2020).

Characteristic	*N*/median (%/range)
Age (median/range)	78 (58–88)
GOLD spirometric stage^a^	
1–2 (mild to moderate)	4 (21%)
3–4 (severe to very severe)	14 (74%)
Physical frailty (SPPB) category (European Medicines Agency, 2018) at initial assessment	
Pre-frail	6 (32%)
Frail	13 (68%)
Long-term oxygen therapy	1 (5%)
Number of comorbidities^b^	2 (0–5)
Gender	
Female	10 (53%)
Male	9 (47%)
Education	
Left school age 15 years or younger	9 (47%)
Left school age 16–19 years	7 (37%)
Post-secondary or university qualifications	3 (16%)
Ethnicity	
Asian, Black or Mixed	3 (16%)
White British/Irish	16 (84%)
Smoking history	
Current smoker	3 (16%)
Ex-smoker	15 (79%)
Never smoked	1 (5%)
Lives alone	11 (58%)
Did not start/complete PR programme^c^	9 (47%)

SPPB: short physical performance battery; PR: pulmonary rehabilitation.

a *n* = 1 missing from PR notes.

b Most commonly reported comorbidities included arthritis, asthma, atrial fibrillation, and falls.

c *n* = 4 did not start, *n* = 5 did not complete.

### Social connection supports COPD self-management

Participants’ accounts showed how social connections with family, friends and neighbors provided sources of emotional, instrumental, and informational support, which impacted positively on self-management.

Emotional support came from both the perceived presence of available social connection, and the actual behaviors of engaging socially with others. The perceived presence of sufficient social resource could reduce anxiety and improve confidence in participants’ ability to respond to health crises. In many cases, knowing someone was there if needed was a huge source of reassurance.

 I’m very lucky. Because if I didn’t have the people around me, then I would worry. (P011)

So I’ve got a back-up. As I say, my daughter, my granddaughters, my grandsons, they’re all there if I need them. I’ve only got to phone them up and they’re there. (P017)

More active influences of social connection were also noted, from social connections providing motivation and disrupting periods of boredom, to explicit encouragement to participate in health behaviors or engage with their local community.

 Somebody’s got to take control, and in my case I find it’s my nephews and nieces who phone up and say, ‘Oh, it’s so-and-so’s birthday, or we’re going here, come with us.’ It brings me out. You just need, every now and again, time to break up the monotony. (P003)

My daughter said, ‘You’ve got to get out Mum, get out a bit more.’ Then when I felt okay, I got my confidence back. I thought, ‘Yes, I’m going to do that.’ (P002)

Practical support via social connections was also depicted as an important element of managing the challenges of living with COPD. Participants described how family, friends, and neighbors might routinely help with instrumental activities of daily living, such as cleaning or shopping. Such support would often be intensified during periods of worsening symptoms or exacerbations, where support might additionally include facilitating access to healthcare services (e.g. through practical assistance or advocacy) or supporting more basic activities of daily living (e.g. cooking, showering).

 Well, yes, my family is pretty good. My boy, as I said, if I want something lifting or doing, he’s there and he’ll do it. . . . My wife does everything for me, really. (P018)

[My daughters] would come around more often, make sure that I’m eating, and I’m drinking, and I have plenty of fluids, and they keep an eye on my temperature and that. (P011)

In some instances, these supportive efforts could also include discouraging engagement in activities; particularly in protective efforts to preserve the participant’s energy, or where there were concerns that the participant’s expectations were not realistic.

But now my son has come back to live here and, bless him, he does quite a lot of the things and I get up and I say, ‘Come on, I’ll do that,’ and we’ve got this little thing, ‘Mum, go and sit in the corner.’ (P009)

It was [my son] that said, ‘Well, are you mad, Mother?’ I said, ‘Why?’ He said, ‘Twice a week you’re going to get up and go out [for pulmonary rehabilitation]?’ (P015)

Participants with connections that were local, reliable, and flexible to changing needs over time seemed to particularly benefit from the role of social connection in self-management. Often this was due to the person with COPD needing support to travel, but sometimes it was due to them needing to prioritize spending their energy on activities of daily living.

 But as I say, looking after myself takes me all my time. (P008)

Where social connections were more distant, less reliable, or simply absent, this could leave gaps in people’s ability to stay engaged with their community and activities of interest. In a few instances, lack of available support could motivate persistence in trying to do activities independently as there was no other option. However, this persistence might be accompanied by feelings of despair or abandonment.

Nobody is ever going to help her be able to go outside on her own and get on a bus anymore because she can’t walk anywhere to be able to do that, really (C013)

You’re on your own most of the time, you feel things that you know you can’t do yourself, but you’ve got to keep trying, that’s what you can do. Don’t know what else to do. But nobody seems to care. (P004)

While generally advantageous, a significant challenge relating to social connection was feelings of becoming a burden. Several participants relayed concerns of becoming a burden on others, and the uncertainty of how they might continue to manage with their advancing condition. This reflected how the nature of their social connections sometimes changed, feeling more functional than fun, or based on reliance rather than reciprocity. This could also evoke strong feelings around what level of support from others would be acceptable to them, potentially reflecting underlying societal or cultural expectations of what is “reasonable” to ask of, or expect from, social connections:

It’s as though I am going to become a liability. At the moment I can manage to just about cope. But if I become a bit of a liability that’s going to be another bit of a problem. As I say, the only thing I’ve got is my godson, but of course he’s got his own family. (P008)

Well, I think it will get worse, love. I’m not being morbid. Then, I don’t want to be here, love, if I get worse, because I’ll be a burden on anyone else in the way I feel (P007)

### The “triple threat” of COPD to social connection

While the potential value of social connections increased in importance with advancing illness, they also became increasingly difficult. Participants’ descriptions illustrated a “triple threat” of COPD and its accompanying symptoms to social connection, with disruption manifesting through physical restrictions, psychological reactions, and societal unawareness.

Participants noted how the physical restrictions imposed particularly by breathlessness, cough, and fatigue, increasingly stifled social participation. This could range from limiting input into conversations, to being unable to maintain a physical presence in their community. As their health worsened, it could become increasingly difficult to keep up with activities with friends and family members. This brought a difficult tension between feeling included and feeling like they were limiting the experience of others.

 Sometimes, you just feel so cut off because you haven’t got enough air in your lungs to join in the conversation. (P019)

I always think, ‘Well, I don’t want the kids hanging around waiting for me,’ and the grandkids. I’ve got *five* grandkids. ‘We can’t do that. Granddad can’t go on that.’ Sometimes I feel I’m better off, really, not being there and let them enjoy it rather than me be there. That doesn’t mean my kids think that, my *two* daughters. They go crazy if they think I’m not coming because I feel that I’m slowing them up, but they don’t understand how I feel when I want them to go off and enjoy themselves (P018)

Psychological responses around anticipated negative outcomes would also lead to avoidance of some activities or encounters. This included anticipated negative experiences directly related to illness and symptoms (e.g. fear of becoming too breathless, fear of catching an infection) and their consequences (e.g. unwanted attention from others). For many, these anticipated consequences were based on previous experiences, such as a member of the public commenting on their cough or breathlessness.

I don’t go out anymore. I used to like to go to have a drink and things. I don’t do that anymore because of my breathing. I’ll walk a little bit, and then my breathing gets really bad, and then people say, ‘Are you alright?’ And that really. . . I don’t want people coming out asking me. Just leave me alone. Let me get on with it (P011)

Sometimes however, the anticipated consequences depicted a broader sense of a lack of safety in their local community. For example, one participant described hesitancies around using portable oxygen in the community, citing fears it might be stolen, and concerns that people might adjust the settings if they saw he was particularly breathless:

They’ve got the things they’ve got in the park just opposite where I live. I couldn’t go there because, again, if I had a problem there, more than likely, someone would just turn that up. ‘Oh, I know what it is. Mate, you need more oxygen. Turn it up, turn it up.’ Plus, being in [town]. . . If I came to it would already by down the road on somebody else’s back. So that’s out the window. (P012)

Participants’ potential sense of disconnection or “otherness” could be further compounded by a perceived lack of public understanding about lung conditions. This included a lack of understanding of what symptoms like breathlessness feel like, but also of the unpredictability of the illness. Some environments people encountered acted as a physical manifestation of this lack of understanding. For example, one participant described her frustration at attending a support service for people with lung conditions, and finding it required attendees to climb a steep set of stairs.

Unless you’ve got the condition, and you’re actually going through it, you really don’t understand. . . . (P014)

one thing I went to attend *three* years ago, *two* years ago, they did a course for people with COPD, or breathing difficulties, in [local town]. It was, again, in a doctor’s surgery. But I had to walk up. . . Well, everybody had to walk up *two* flights of stairs, which I actually thought was a bit of a no-no for people with breathing difficulties. (P015)

It was therefore unsurprising that some participants particularly appreciated the opportunity to connect with others with similar conditions, for example through pulmonary rehabilitation, where they felt more fully understood.

### The inseparable nature of social health

The importance of maintaining social connection despite increasing difficulties was further illustrated by the inseparable nature of social health from participants’ physical and psychological health. This could include contributions of social connection to mental health via influences on wellbeing, but also on physical health via influences on participation.

In some cases, these domains were so intertwined that bidirectional relationships between social disconnection and poor health could progress in vicious, sometimes intersecting, cycles. For example, one participant described a period where her poor physical health was contributing to low mood, and with a lack of social connection she felt unable to motivate herself to be active, which then contributed to further psychological challenges. These cycles could be gradual, but sometimes were accelerated by health events like exacerbations, falls, or flare-ups of comorbid conditions.

But when you haven’t got the energy, you feel so low and you can’t do it. You don’t want to do it. If I had somebody there to try to help me to motivate myself, maybe I would have. But when you feel so low, you cannot- If the chest is really bothering you, you don’t want - you try to breathe, and you cannot breathe. That’s painful. So those days, I just lie there with so many things going through my mind. (P005)

Social connection also however presented an opportunity to disrupt these cycles, whether through connection with family members or engaging in activities in their wider community. Social connections could contribute to their wellbeing, in turn improving self-confidence and motivation to engage in future activities. Social connections could also support participation in activities or actions that contribute to the person’s physical capacity, whether by providing prompt response to exacerbations, or supporting their participation in social activities and hobbies that involve a level of physical activity.

Yes, I think I’m depressed, and I’ve been wondering whether I should go over and tell the doctor. I get very. . . Because I’ve got no energy. That’s the thing, I’ve got no energy. And yet, when I’ve been away and when I go to [my son’s], I pull out the reserves of energy, so maybe I need to do that a lot more. (P015)

I go to a day centre once a week, just to get out of the flat. And going to [Hospital 2] for an injection helps me to get out of the house. I’m seeing a bit of the world and mixing with people and things. Because if you tend to stay in, you get a bit isolated, so it’s important to get out and meet people and socialise, and that sort of thing. (P006)

At their best, these pathways could even transform into virtuous cycles, whereby social connection and its positive impacts could lead to further increased social engagement and potential health benefits. For example, one participant described how a positive experience of attending a local community group on a Friday led her to hear about and join another local chair exercise group on a Monday. Health contacts had potential to be an important facilitator of these opportunities, either through the interventions they provided or through signposting elsewhere.

At the Friday club there were a couple there and they said, ‘Have you heard about the chair exercise in the library?’ I said, ‘No, not really.’ She said, ‘Every library has them.’ I went into the *one* at the bottom and he said, ‘Yes, all you’ve got to do is put your name down and it costs £1 every Monday morning.’ (P002)

Talking to *one* of the guys down there, he said, “Yes, I know exactly what you mean. [Pulmonary rehabilitation is] like going on your holiday, isn’t it? You haven’t got to sit there staring at that wall. (P014)

While participants’ stories highlighted these opportunities to disrupt negative cycles and prompt more positive ones, they also illustrated how these opportunities were sometimes limited by social and environmental factors. This ranged from the cost and accessibility of transport, to not feeling welcome in some spaces, to a lack activities provided in their local area.

But as it says in *one* of my magazines, try not to go to the gym a lot when you get older because there are always stacks of young girls or young chaps and they’re always pounding around, aren’t they, on their machines and what not. It really puts you off a bit. (P010)

But in this area there’s *one* class at [neighbouring area] which is over-subscribed, *1000*%. We’re not in [neighbouring area], we’re in [here]. There aren’t any services and I think that, unfortunately, is where the councils do fall down (P003)

**Figure 1.**
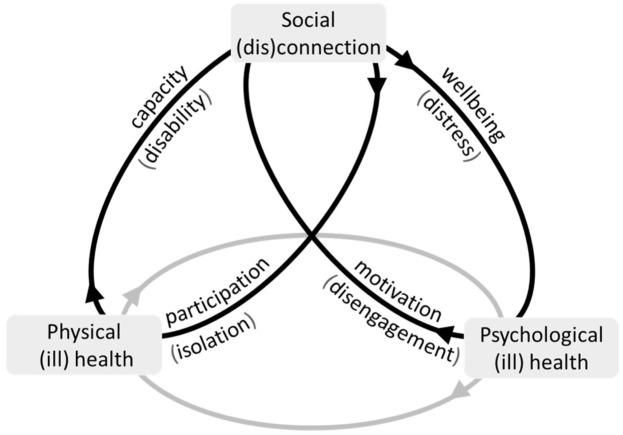
Intersecting cycles model of how social (dis)connection may contribute to physical and psychological health.

The relationships between social health with physical and psychological domains, including potential contributing mechanisms, can be summarized in an intersecting cycles model (Figure 1). This depicts how social disconnection can form part of vicious cycles featuring distress, disengagement, isolation, disability, but also how social connection can conversely contribute to virtuous cycles of wellbeing, motivation, participation, and increased physical capacity.

## Discussion

In exploring the role of social connection in living with COPD, this analysis sheds light on an often-neglected area, with implications both within and maybe beyond respiratory research and care. Participants described how social connections provide several layers of emotional and practical support which can advantage self-management when living with COPD. This can include more explicit forms of support and encouragement, but also more implicit benefits such as feeling reassured that support is available when needed. However, people with COPD also experience a “triple threat” of respiratory related disruptions to their social connections, in the form of physical restrictions, psychological reactions, and societal unawareness relating to their illness and symptoms such as breathlessness. Together, these experiences culminate in social connection becoming increasingly valuable, yet increasingly difficult, as disease progresses. The inseparable nature of social health from wider physical and psychological health came through strongly in participants’ stories. As depicted in our intersecting cycles model, at its worst, social disconnection can contribute to vicious cycles of distress, disengagement, isolation, disability. However, intervening to disrupt or change these cycles, including via social connection, has potential to prompt virtuous cycles of wellbeing, motivation, participation and increased physical capacity.

Our findings align with the challenges described elsewhere by people with COPD (Clari et al., 2018; Disler et al., 2014), but add further depth about their lived experience of social connections. Existing work describing dyspnea invisibility has a strong relevance here (Gysels and Higginson, 2008; Serresse et al., 2022), whereby people living with chronic breathlessness describe how this symptom can be invisible and little understood, contributing to feelings of loneliness. While our participants also highlighted the role of respiratory symptoms like cough, breathlessness tends to be a central theme of how COPD disrupts social connection, either directly or via impacts on thoughts and behaviors. People with COPD have previously reported breathlessness as their main barrier to social participation (Evangelista et al., 2021; Michalovic et al., 2020). The interrelatedness of breathlessness and social connection may have implications for the concept of “breathing space” (Hutchinson et al., 2018); a person’s capacity to live with chronic breathlessness. While domains affecting breathing space are suggested to include an individual’s coping, help-seeking, and professionals’ responses, the growing evidence around the role of social dimensions may suggest a role for social connectedness as another important dimension.

Our integrative approach provides a novel lens on the bidirectional relationships between different domains of health, including the role of social connection as part of intersecting cycles. Our findings align with Holt-Lunstad’s model linking social connection and health outcomes, including the cyclical nature of these relationships (Holt-Lunstad, 2021), illustrating how they may manifest in a respiratory context. This includes the “triple threat” of how respiratory-related symptoms like breathlessness can disrupt social connection through physical, psychological, and social mechanisms. The challenges around respiratory symptoms are not limited to COPD, and other symptoms (e.g. pain, fatigue) may have comparable impacts in other long-term conditions. However, our findings do highlight a population with a particular risk of disrupted social connection. Such challenges are likely further compounded when considered alongside what is known about the detrimental impacts of smoking-related stigma (Brighton et al., 2022; Woo et al., 2021). Quantitative modeling to determine the magnitude and temporal nature of some of these relationships may provide the key to successful interventions to support social connection and unlock wider health benefits.

On an individual level, our findings indicate the centrality of attending to social health within clinical encounters, or “at the epicentre of actions” (Bouloukaki et al., 2024). This might range from awareness that those lacking in social connection may be in a more precarious position when health events occur, to exploring the social impacts of COPD with individuals and supporting continued engagement in activities that are important to them. There may be a role here for strengthening links between respiratory services and social prescribing initiatives (Bickerdike et al., 2017); learning from pilot work where individuals with COPD have been supported to connect with local activities (Welch et al., 2020). Building on existing opportunities, for example maximizing on social elements of group-based pulmonary rehabilitation, may also be valuable. However, interventional work relating to the acceptability and effectiveness of interventions to improve social outcomes for people with COPD are limited to date and require further study (Brighton et al., 2022). This is not about medicalizing social connection, rather acknowledging the interconnectedness of social, physical, and mental health (Aravantinou-Karlatou et al., 2023; Mete, 2024), and recognizing that clinicians may have another tool at their disposal in the form of social connection.

On a more societal level, participants’ stories illustrate how a lack of public understanding of respiratory conditions and limited community resources can contribute to further isolation of people with COPD. This rings true with calls to attend to the role of “lonelygenic environments”: the ways in which social and built environments can cause or aggravate loneliness, and the importance of congruence between personal and place circumstances (Feng and Astell-Burt, 2022). It also further substantiates calls to align our environments to our aging population (Department of Health and Social Care, 2023), and highlights the need for more awareness-raising of respiratory illness and symptoms. This work could learn from the success of “Dementia Friendly Communities”: an initiative to support people, processes, and places to be more respectful and accessible to people living with dementia (Buckner et al., 2019; Darlington et al., 2021).

This study has several strengths and limitations. The nature of this secondary analysis means that the interview topic guide was not specifically designed to explore social connections, and therefore some areas of interest may not have been covered in depth. For example, challenges around sex and intimacy (Farver-Vestergaard et al., 2022), financial difficulties (Lee et al., 2019), and stigma (Woo et al., 2021), are all sensitive topics which may not be raised if not specifically asked about. However, the level of discussion of social connection despite this speaks to the prominence of this aspect of participants’ lived experience. Similarly, due to the original aims, sample for this study is limited to people referred for pulmonary rehabilitation. Given the importance of social connection in living with COPD illustrated by our participants, research designed specifically to understand how we can support people with COPD to maintain connections with their communities as their illness progresses is needed. Purposive sampling did ensure inclusion of diverse experiences within this cohort, particularly in terms of level of frailty and varying engagement with rehabilitation (including non-attendance). While some of our data indicated potential social and cultural influences in relation to the expectations of the roles of relatives and friends in supporting living with COPD, further work with more culturally diverse samples would allow this aspect to be considered in more depth. As participants lived with multiple conditions alongside their COPD and experienced a range of common, distressing symptoms (e.g. breathlessness, fatigue), our findings may be applicable to other long-term conditions. Input from people affected by COPD as well as professionals with diverse backgrounds strengthened the credibility and rigor of our analysis and interpretation. We also worked with existing theoretical approaches to social connection to deepen our understanding of the data. However, as our approach was limited to social connections between people, there may be benefits to future work exploring other forms of social connections (e.g. with pets).

Overall, our findings illustrate how social connections can become increasingly valuable, yet increasingly difficult to maintain, as COPD progresses. The numerous advantages of social connection to self-management, and the inseparable nature of social health from wider physical and psychological wellbeing, suggest this domain requires attention and support as part of a truly biopsychosocial approach to respiratory research and care. In addition to supporting individuals with challenges around social connection, societal initiatives that address stigma, public understanding, and accessible environments will also be important in supporting people with COPD to stay connected with their communities. Given the diverse comorbidities and range of symptoms experienced by our participants, it is likely the implications of this work extend beyond respiratory illness.
